# Absence of Mutations in Exon 6 of the TARDBP Gene in 207 Chinese Patients with Sporadic Amyotrohic Lateral Sclerosis

**DOI:** 10.1371/journal.pone.0068106

**Published:** 2013-07-09

**Authors:** Cheng-hui Ye, Xi-lin Lu, Min-ying Zheng, Jun Zhen, Zhi-Ping Li, Lei Shi, Zhi-yong Liu, Lu-yang Feng, Zhong Pei, Xiao-li Yao

**Affiliations:** 1 Department of Neurology, The First Affiliated Hospital, Sun Yat-sen University, Guangzhou, China; 2 Department of Rehabilitation Medicine, The Fifth Affiliated Hospital, Sun Yat-sen University, Zhuhai, Guangdong, China; 3 Department of Respiratory Medicine, The First Affiliated Hospital, Sun Yat-sen University, Guangzhou, China; 4 Department of Geriatrics, The First Affiliated Hospital, Sun Yat-sen University, Guangzhou, PR China; 5 Guangdong key laboratory for diagnosis and treatment of major neurological diseases, Guangdong, China; 6 National Key Clinical Department, Guangzhou, China; 7 National Key Discipline, Guangzhou, China; University of Florida, United States of America

## Abstract

Mutations in the TARDBP gene, which encodes the Tar DNA binding protein, have been shown to causes of both familial amyotrophic lateral sclerosis (FALS) and sporadic ALS (SALS). Recently, several novel TARDBP exon 6 mutants have been reported in patients with ALS in Europe and America but not in Asia. To further examine the spectrum and frequency of TARDBP exon 6 mutations, we investigated their frequency in ethnic Chinese patients with sporadic ALS. TARDBP exon 6 was screened by direct sequencing in 207 non-SOD1 SALS patients and 230 unrelated healthy controls but no mutations were identified. Our data indicate that exon 6 mutations in TARDBP are not a common cause of SALS in Han Chinese population from Southern Mainland China.

## Introduction

Amyotrophic lateral sclerosis (ALS) is a devastating, rapidly progressive, fatal neurodegenerative disorder that develops as a combination of upper and lower motor neuron symptoms. ALS patients have a very short median survival duration ranging from three to five years and always die from respiratory failure. Most (90%) ALS cases are sporadic ALS (SALS), while 5–10% are familial amyotrophic lateral sclerosis (FALS), and SOD1 mutations account for 20%–25% of FALS case. TARDBP gene, was discovered in 2006 as the second gene associated with ALS. It is located on chromosome 1p36.21, contains six exons, and encodes TAR DNA-binding protein 43 (TDP-43), a 43-kDa highly conserved heterogeneous ribonucleoprotein (RNP) involved in specific pre-mRNA splicing and transcription events. It was first described as a regulator of HIV-1 gene expression [1], and is now regarded as an important disease protein in ALS where it forms a major component of ubiquitinated motor neuron inclusions [2].

To date, more than 40 different TARDBP mutations have been described [3], the most of which are missense substitutions. The majority of mutations identified in ALS patients are located in exon 6, with the exception of D169G mutation [4]. Although TARDBP mutations have been extensively studied in Caucasian populations, limited studies have been conducted on the TARDBP mutations in Chinese population. In the present study, therefore, we investigated the distribution and frequency of mutations in TARDBP exon 6 in SALS patients of Han Chinese descent patients from a cohort consisting of 207 SALS patients and 230 unrelated healthy controls. Our findings suggest that exon 6 mutations in TARDBP are not a common cause of SALS in Han Chinese population from Southern Mainland China.

## Materials and Methods

### Subjects

This study included 207 ethnic Chinese subjects diagnosed with SALS(135 men and 72 women) and 230 age- and sex-matched healthy control subjects. ALS patients were recruited from the Department of Neurology of the First Affiliated Hospital, Sun Yat-sen University, China. All cases and controls in the study were recruited from Southern Mainland China between March 2003 and November 2011.

Control samples were of a similar ethnic origin who with no reported history of neurological disorders. Clinical diagnosis was performed according to the El Escorial revised criteria by two senior physicians [5]. The project was approved by the Ethics Committee of the First Affiliated Hospital of Sun Yat-sen University. All subjects gave written informed consent for the participation of the study. DNA was extracted from leukocytes in peripheral blood of the subjects.

### Mutation Detect

Genomic DNA from patients and controls was isolated from peripheral blood according to standard protocols. The coding region of TARDBP exon 6 was analyzed using primer combinations based on TARDBP intronic sequences [6] PCR was carried out by mixing 20 pmol of each primers and 20 ng of genomic DNA template with 1.25u TaKaRa Taq, 5 µl 10×PCR Buffer (Mg 2+ Plus), and 4 µl 2.5 mM dNTP Mixture (Millipore Chemicon) in a final volume of 25 µL. The PCR was carried out using a BIOMETRA amplification system at 94°C for 2 min then 35 cycles of 94°C for 30 s, 63°C for 45 s and 72°C for 80 s. After a final elongation step at 72°C for 10 s, PCR products were stored at 4°C and run on ABI 3730 Automated DNA Sequencer according to previously published methods [7]. Sequences obtained by sample sequencing were compared with the published TARDBP genomic DNA sequence (NCBI Sequence Viewer NT_021937.18).

## Results

Mutational detection found that none of the sporadic ALS or the age-, sex-, and ethnicity-matched healthy controls carried mutation in exon 6 of TARDBP. The demographics and clinical characteristics of the ALS patients in the present study agreed with previous reports [8]. The average age of symptom onset was 51 years (SD 51.35±11.28). 176 patients described spinal-onset disease and 31 patients presented with bulbar-onset disease.

## Discussion

More than 40 dominant mutations in TARDBP exon 6 have previously been reported in both sporadic and familial ALS cases [3], [9]. In different Chinese populations,four substitution variants, p.S292N [6], p.G298S [10], p.M337V and p.N378D [8] were previously found in FALS subjects and four variants, S292N, G348V, A366A [3] and p.N378S [11] were detected in SALS cases. Those TARDBP variants are located in exon 6, but their pathological role has not yet been established because of small sample size of these studies. TARDBP mutation frequencies differ between populations of different ethnicity. For example, the frequency of TARDBP mutations in the Asian population is lower than in European population. Additionally, the frequency of TARDBP mutations in all ALS cases is lower in the Asian population (Chinese SALS patients, 0.73%; Japanese patients, 0.29%) than the European population (1–3%) [3], [12].

**Figure 1:**
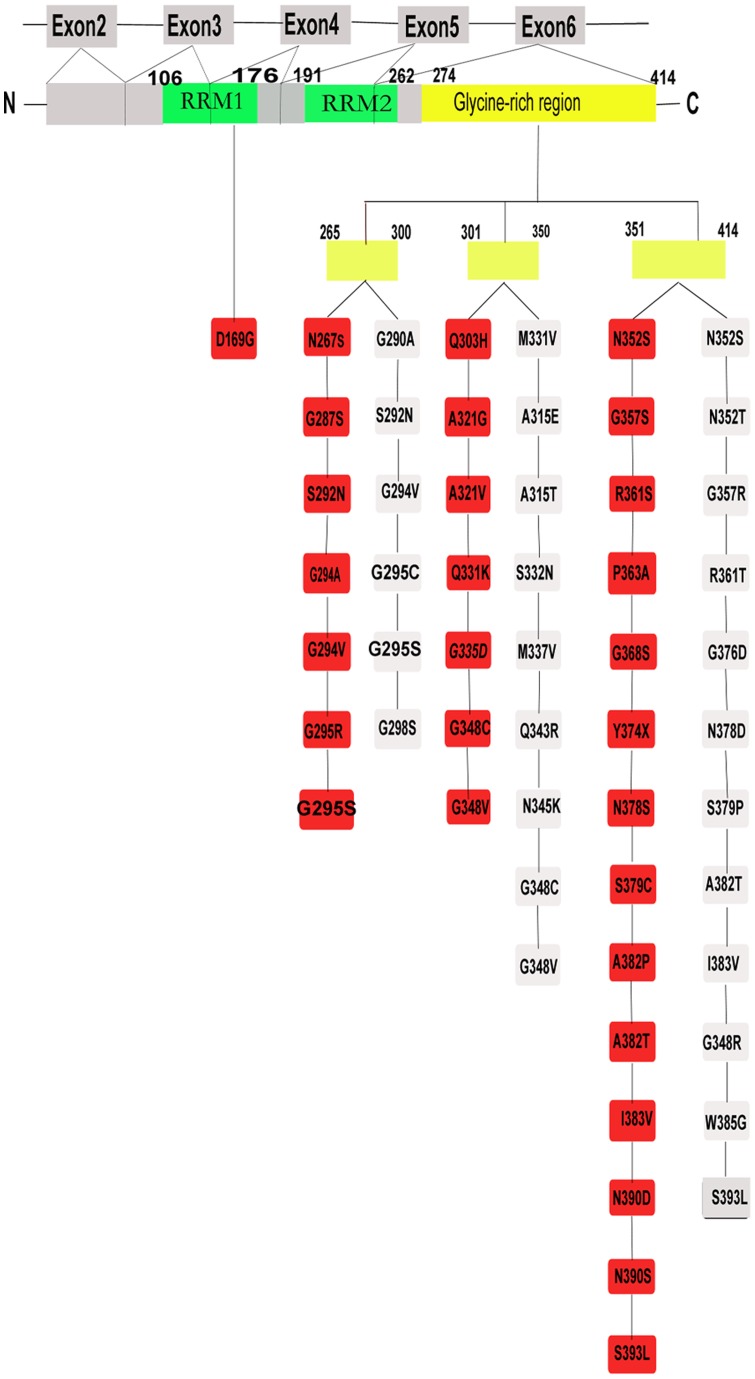
TDP-43 Mutations in ALS. TDP-43 have been identified in sporadic (red) and familial (**grey**) ALS patients. C = carboxyl, N = Nuclear, RRM = RNA-recognition motifs.

In this study, we screened a cohort of 207 SALS and 230 normal controls for mutations in exon 6, but found no novel variant and did not identify any known pathogenic mutations. The majority of TARDBP mutations identified in ALS patients are located in exon 6, with the exception of the D169G mutation that is located in RNA-recognition motif 1 (**Figure 1**). There are several limitations in the present study. First of all, we only screened the exon 6 for TARDBP mutations because the majority of TARDBP mutations are located in exon 6. Recently, D169G mutation was detected in TARDBP exon 4 in France. Although D169G mutation has not been detected in Asia, further study is needed to screen all TARDBP exons for potential novel Chinese TARDBP mutation outside exon 6. In addition, mutations in TARDBP have been linked to both familial and sporadic patients. However, the mutations in TARDBP occur in sporadic cases at a much lower rate. Therefore, the present study may not be robust enough to detect rare mutations because of relative small number of ALS patients. Further study with larger cohort of patients is required to address this issue. Although our sample size is small and may not therefore be fully representative, our results, together with previous reports, suggest that exon 6 mutations in TARDBP are not a common cause of SALS in Han Chinese population from Southern Mainland China [13].

The cause of ALS remains somewhat unclear, but genetic mutations are increasingly detected in sporadic ALS patients, suggesting that genetics plays a more important role than previously thought. However, as 95% of ALS cases occur with no family history, non-genetic factors such as environmental exposures should still be considered as the major contributory factor in SALS cases. Further study is necessary to screen all TARDBP exons for a potential novel Chinese TARDBP mutation outside exon 6.
